# Activating transcription factor 5 enhances radioresistance and malignancy in cancer cells

**DOI:** 10.18632/oncotarget.2912

**Published:** 2015-02-17

**Authors:** Seiichiro Ishihara, Motoaki Yasuda, Akihiro Ishizu, Masayori Ishikawa, Hiroki Shirato, Hisashi Haga

**Affiliations:** ^1^ Faculty of Advanced Life Science, Hokkaido University, Kita-ku, Sapporo 060–0810, Japan; ^2^ Research Center for Cooperative Projects, Graduate School of Medicine, Hokkaido University, Kita-ku, Sapporo 060–8638, Japan; ^3^ Department of Oral Pathobiological Science, Graduate School of Dental Medicine, Hokkaido University, Kita-ku, Sapporo 060–8586, Japan; ^4^ Division of Medical Laboratory Science, Faculty of Health Sciences, Hokkaido University, Kita-ku, Sapporo 060–0812, Japan; ^5^ Department of Medical Physics, Graduate School of Medicine, Hokkaido University, Kita-ku, Sapporo 060–8638, Japan; ^6^ Department of Radiology, Graduate School of Medicine, Hokkaido University, Kita-ku, Sapporo 060–8638, Japan

**Keywords:** Activating transcription factor 5, radioresistance, cell growth, cell invasion, cancer cells

## Abstract

Radiotherapy is effective for treating various types of tumors. However, some cancer cells survive after irradiation and repopulate tumors with highly malignant phenotypes that correlate with poor prognosis. It is not known how cancer cells survive and generate malignant tumors after irradiation. Here, we show that activating transcription factor 5 (ATF5) promotes radioresistance and malignancy in cancer cells after irradiation. In the G1-S phase of the cell cycle, cancer cells express high levels of ATF5, which promotes cell cycle progression and thereby increases radioresistance. Furthermore, ATF5 increases malignant phenotypes, such as cell growth and invasiveness, in cancer cells *in vitro* and *in vivo*. We have identified a new mechanism for the regeneration of highly malignant tumors after irradiation and shown that ATF5 plays a key role in the process.

## INTRODUCTION

Radiotherapy is effective for treating various types of tumors [1]. However, some cancer cells survive after irradiation and repopulate tumors with highly malignant phenotypes, such as high proliferative ability and invasiveness, which correlate with poor prognosis [2–5]. As shown in our previous studies, lung cancer cells that survive irradiation acquire invasive ability that is dependent on cell-matrix adhesion regulated by integrin β1, cellular contractile force modulated by myosin regulatory light chain (MRLC), and molecular signaling mediated by epidermal growth factor receptor and other molecules [6–8]. However, it is not known how cancer cells survive and generate highly malignant tumors after irradiation.

Activating transcription factor 5 (ATF5, also referred to as ATFx) is a member of the ATF/cAMP response element-binding family of transcription factors [9–11]. ATF5 regulates the differentiation of neural progenitor cells and adipose-derived stem cells [12, 13]. Moreover, ATF5 is expressed at higher levels in neural and breast cancer cells than in the respective non-cancerous cells [14, 15]. Repression of ATF5 induces apoptotic cell death in neural and breast cancer cells but does not kill non-transformed cells. Our group previously reported that ATF5 increases radioresistance and motility in mouse fibrosarcoma cells [16]. These findings suggest that ATF5 is a key molecule for cancer cell survival and malignancy after irradiation. Therefore, in this study, we investigated the role of ATF5 in radioresistance and in the development of malignant phenotypes in human cancer cells after irradiation.

## RESULTS

### ATF5 enhances radioresistance

**Figure 1:**
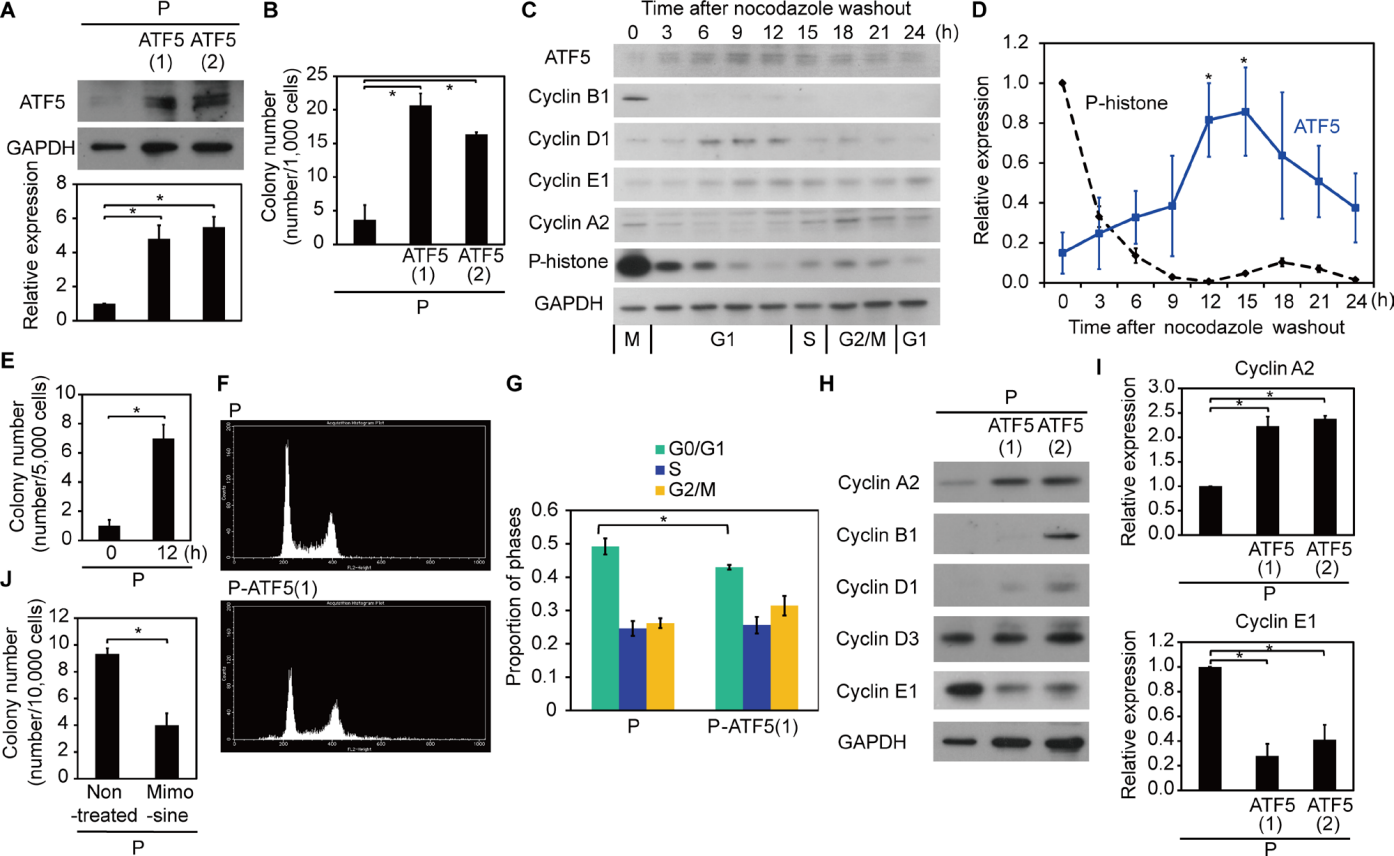
ATF5 enhances radioresistance by promoting cell cycle progression **(A)** Western blot of ATF5 and GAPDH. The graph shows the relative expression of ATF5. P: subclonal A549 cells. P-ATF5(1), (2): subclonal P cells overexpressing ATF5. **(B)** Colony number after irradiation. **(C)** Western blot of ATF5, cell cycle-regulated genes, and GAPDH in synchronized P cells. The numbers indicate the time after nocodazole washout. **(D)** Relative expression of ATF5 and P-histone in C. **(E)** Colony number of P cells after irradiation. The cells were irradiated after cell cycle synchronization. The horizontal axis indicates the time after nocodazole washout. **(F)** Flow cytometry of cells stained with propidium iodide. **(G)** Relative proportion of cells in the cell cycle phases determined by F. Error bars = s.e.m. from 3 (P) or 4 (P-ATF5(1)) independent experiments. **(H)** Western blot of cyclin and GAPDH. **(I)** Relative expression of cyclin A2 and cyclin E1 in H. **(J)** Colony number of P cells treated or not treated with mimosine after irradiation. *P < 0.05. Error bars = s.e.m. from 3 independent experiments except G.

First, we investigated whether ATF5 regulates radioresistance in human cancer cells. We transfected the *ATF5* gene into subclonal A549 human lung adenocarcinoma cells (P) and established subclonal P cells overexpressing ATF5 (P-ATF5(1) and P-ATF5(2) cells). ATF5 overexpression in these cell lines was confirmed with western blotting (Figure 1A). We then evaluated radioresistance in the cells by counting the number of colonies generated by cells that survived irradiation. The colony number in P-ATF5(1) and (2) cells 12 days after 10-Gy irradiation was greater than that in P cells (Figure 1B). The number of colonies after irradiation did not differ remarkably between P and subclonal P cells overexpressing AG-CAAX (P-CAAX was the negative control; Supplementary Figure S1A, S1B). On the other hand, the colony number 12 days after seeding in the absence of irradiation was similar in the three cell lines (Supplementary Figure S2A). Colony formation by P and P-CAAX cells was similar under non-irradiated conditions (Supplementary Figure S1C). These results indicate that ATF5 enhances radioresistance but does not regulate colony formation itself in A549 lung adenocarcinoma cells.

### The cell cycle decides ATF5 expression

Next, we determined whether ATF5 was consistently expressed in each cell line and whether ATF5 expression changed under specific conditions. We hypothesized that ATF5 expression varies with the cell cycle because previous reports have indicated that radioresistance changes depending on the cell cycle phase [17–20]. Therefore, we analyzed ATF5 expression in P cells synchronized with nocodazole treatment [21]. After nocodazole washout, the cells expressed cell cycle markers for specific cell cycle phases, indicating that cell cycle synchronization was successful (Figure 1C, 1D and Supplementary Figure S3A): cyclin B1, cyclin D1, cyclin E1, cyclin A2, and P-histone indicated G2-M, G1, G1-S, S-M, and M phases, respectively [22, 23]. ATF5 was highly expressed from late G1 phase to S phase (Figure 1C, 1D and Supplementary Figure S3A). Thus, ATF5 is not consistently expressed but changes according to the cell cycle phase in cancer cells.

Because ATF5 expression was dependent on the cell cycle phase, we next investigated whether radioresistance was dependent on the cell cycle. We compared synchronized cells in late G1 phase (obtained 12 h after nocodazole washout) that displayed high ATF5 expression with synchronized cells in M phase (obtained 0 h after nocodazole washout) that showed low ATF5 expression (Figure 1C, 1D and Supplementary Figure S2). The cells irradiated 12 h after nocodazole washout had higher radioresistance than the cells irradiated 0 h after nocodazole washout (Figure 1E). Colony formation by the two synchronized cell populations was similar under non-irradiated conditions (Supplementary Figure S2B). Thus, ATF5 expression and radioresistance are dependent on the cell cycle in cancer cells.

### ATF5 promotes cell cycle progression

To understand the mechanism underlying radioresistance, we investigated how ATF5 regulates radioresistance. We hypothesized that ATF5 enhances radioresistance via regulation of the cell cycle because ATF5 expression was dependent on the cell cycle (Figure 1C, 1D and Supplementary Figure S3A, S3B, S3C). The proportion of P-ATF5(1) cells in G0/G1 phase was lower than the proportion of P cells in G0/G1 phase (Figure 1F, 1G). In contrast, the proportion of P-CAAX cells in the G0/G1 phase was higher than that in P cells (Supplementary Figure S1D). Combined with results showing that ATF5 was highly expressed during late G1 to S phase (Figure 1C, 1D and Supplementary Figure S3A, S3B, S3C), the finding indicates that ATF5 promotes G1/S transition at late G1 phase. Indeed, P-ATF5(1) and (2) cells expressed high levels of cyclin A2, a marker of S-M phases, and low levels of cyclin E1, a marker of G1-S phases, when compared to P cells (Figure 1H, 1I), whereas there were no remarkable differences in the expression of cyclin A2 and cyclin E1 between P and P-CAAX cells (Supplementary Figure S1E). We then examined radioresistance when the G1/S transition was inhibited with mimosine treatment [24]. Mimosine treatment increased the proportion of G0/G1 phase cells, reduced the number of G2/M phase cells, and suppressed cyclin A2 expression (Supplementary Figure S4A, S4B, S4C, S4D), indicating that the treatment inhibited the G1/S transition. P cells treated with mimosine had lower radioresistance than control cells, whereas there was no significant difference in colony formation by these cells under non-irradiated conditions (Figure 1J and Supplementary Figure S2C). These results show that ATF5 promotes radioresistance by promoting the G1/S transition.

Because ATF5 increased the expression of cyclin A2 and repressed the expression of cyclin E1, we determined whether ATF5 regulated radioresistance via the upregulation of cyclin A2 and/or the downregulation of cyclin E1. Downregulation of cyclin E1 repressed radioresistance in P cells but did not affect colony formation in non-irradiated cells (Supplementary Figure S5A, S5B, S5C). Knockdown of cyclin A2 in P-ATF5(1) cells did not affect radioresistance or affect colony formation in non-irradiated cells (Supplementary Figure S5D, S5E, S5F). Therefore, ATF5 enhances radioresistance independent of cyclin E1 and cyclin A2 expression.

### ATF5 enhances malignant phenotypes

**Figure 2:**
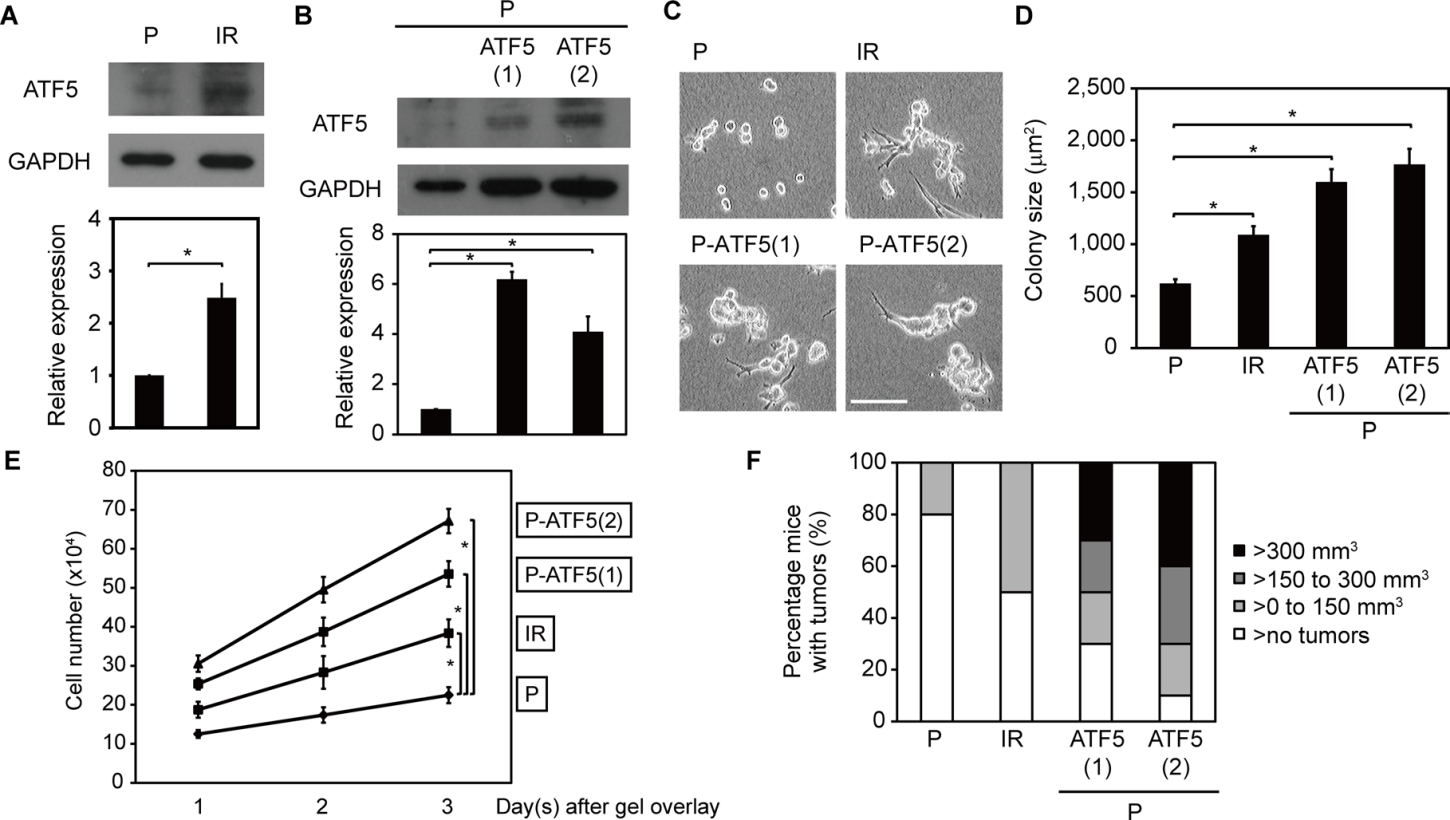
ATF5 promotes cell growth **(A)** Western blot of ATF5 and GAPDH. The graph indicates the relative expression of ATF5. P: subclonal A549 cells. IR: P cells that survived irradiation. **(B)** Western blot of ATF5 and GAPDH. P-ATF5(1), (2): subclonal P cells overexpressing ATF5. **(C)** Phase-contrast images. Bar = 200 μm. **(D)** Colony size of cells in C. Error bars = s.e.m. from 134 (P), 107 (IR), 143 (P-ATF5(1)), or 155 (P-ATF5(2)) colonies in 3 independent experiments. **(E)** Number of cells. The horizontal axis indicates the days after collagen gel-overlay. **(F)** Percentage of mice with tumors. The colors of the bars indicate the tumor volume. *n* = 10 mice. *P < 0.05. Error bars = s.e.m. from 3 independent experiments except D. The cells were cultured in collagen gel-overlay conditions.

Cancer cells that survive after irradiation acquire malignant phenotypes [2–5]. We previously reported that P cells that survive after irradiation (IR cells) are more invasive than non-irradiated P cells [6–8]. Therefore, we examined the phenotypes of P and IR cells to determine whether ATF5 promotes malignancy in cancer cells. Cells were cultured in collagen gel-overlay conditions, enabling us to observe tumor-mimic colony formation and cell invasion in a three-dimensional collagen matrix *in vitro*. In collagen gel-overlay conditions, ATF5 expression was higher in IR cells than in P cells (Figure 2A). Furthermore, the transcriptional activity of cAMP response element (CRE), which is repressed by ATF5 [10], was significantly lower in IR cells than in P cells (Supplementary Figure S6A). In addition, ATF5 repression increased CRE activity in IR cells (Supplementary Figure S6B). These results indicated that ATF5 expression and activity were upregulated in IR cells. ATF5 expression was also higher in P-ATF5(1) and (2) cells than in P cells in collagen gel-overlay conditions (Figure 2B). However, ATF5 expression was similar between P and P-CAAX cells in collagen gel-overlay conditions (Supplementary Figure S1F). In collagen gel-overlay conditions, tumor size and cell number in IR, P-ATF5(1), and P-ATF5(2) cells were significantly greater than in P cells (Figure 2C–2E), whereas cell number in P and P-CAAX cells was similar (Supplementary Figure S1G). Thus, ATF5 enhances the growth of cancer cells *in vitro*.

We also examined the roles of ATF5 in tumorigenesis and cell growth *in vivo*. We analyzed tumor number and size after injecting cancer cells into nude mice. A greater number of mice developed tumors after injection of IR, P-ATF5(1), and P-ATF5 (2) cells than after injection of P cells (Figure 2F). Furthermore, tumor volume was significantly larger in mice injected with P-ATF5(1) and (2) cells than in mice injected with P cells (Figure 2F and Supplementary Figure S7A, S7B). In addition, the proportion of mitotic cells in the tumors was higher after injection of IR, P-ATF5(1), and P-ATF5(2) cells than after injection of P cells (Supplementary Figure S7C). These results show that ATF5 promotes tumorigenesis and cancer cell growth *in vivo*.

**Figure 3:**
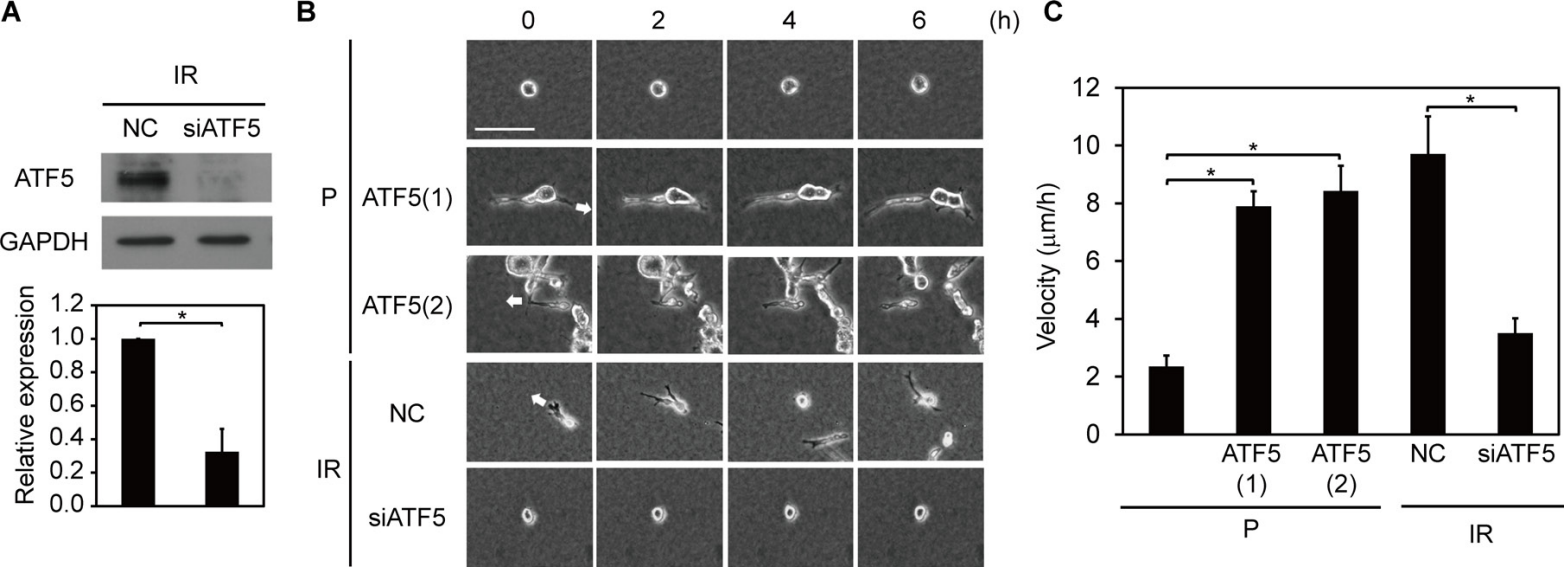
Invasiveness is dependent on ATF5 expression **(A)** Western blot of ATF5 and GAPDH. The graph indicates the relative expression of ATF5. IR: subclonal A549 cells (P) that survived irradiation. NC: negative control cells. siATF5: cells transfected with siRNA targeting ATF5. Error bars = s.e.m. from 3 independent experiments. **(B)** Time-lapse phase-contrast images. The numbers indicate the time from the start of observation. The arrows indicate the direction of cell migration. P-ATF5(1), (2): subclonal P cells overexpressing ATF5. Bar = 100 μm. **(C)** Velocity of cell migration in B. Error bars = s.e.m. from 12 (P), 14 (P-ATF5(1) and (2)), or 15 (IR-NC and siATF5) cells in 3 independent experiments. *P < 0.05. The cells were cultured in collagen gel-overlay conditions.

We also examined the roles of ATF5 in invasiveness *in vitro* and *in vivo*. We downregulated ATF5 expression in IR cells using an RNAi method (Figure 3A) and assessed invasiveness *in vitro* by analyzing migration in collagen gel-overlay conditions. Relative to P and P-CAAX cells, P-ATF5(1) and (2) cells exhibited higher migratory ability with the generation of protrusions (Figure 3B, 3C, Supplementary Figure S1H, S1I, and Videos S1–S3). In addition, ATF5 downregulation suppressed invasiveness in IR cells (Figure 3B, 3C and Videos S4, S5). Furthermore, tumors generated by IR, P-ATF5(1), and P-ATF5(2) cells in nude mice displayed an invasive front on the border between tumor tissue and normal tissue, whereas tumors generated by P cells did not (Supplementary Figure S7D). Therefore, ATF5 enhances invasiveness in cancer cells *in vitro* and *in vivo*.

Previous studies have reported that suppression of ATF5 induces apoptosis in cancerous cells [14, 15]. However, in this study, downregulation of ATF5 did not trigger apoptosis in IR cells (Supplementary Figure S6C). This result suggests that the role of ATF5 in apoptosis induction in cancer cells is dependent on the cell type.

### ATF5 induces integrin β1 expression

**Figure 4:**
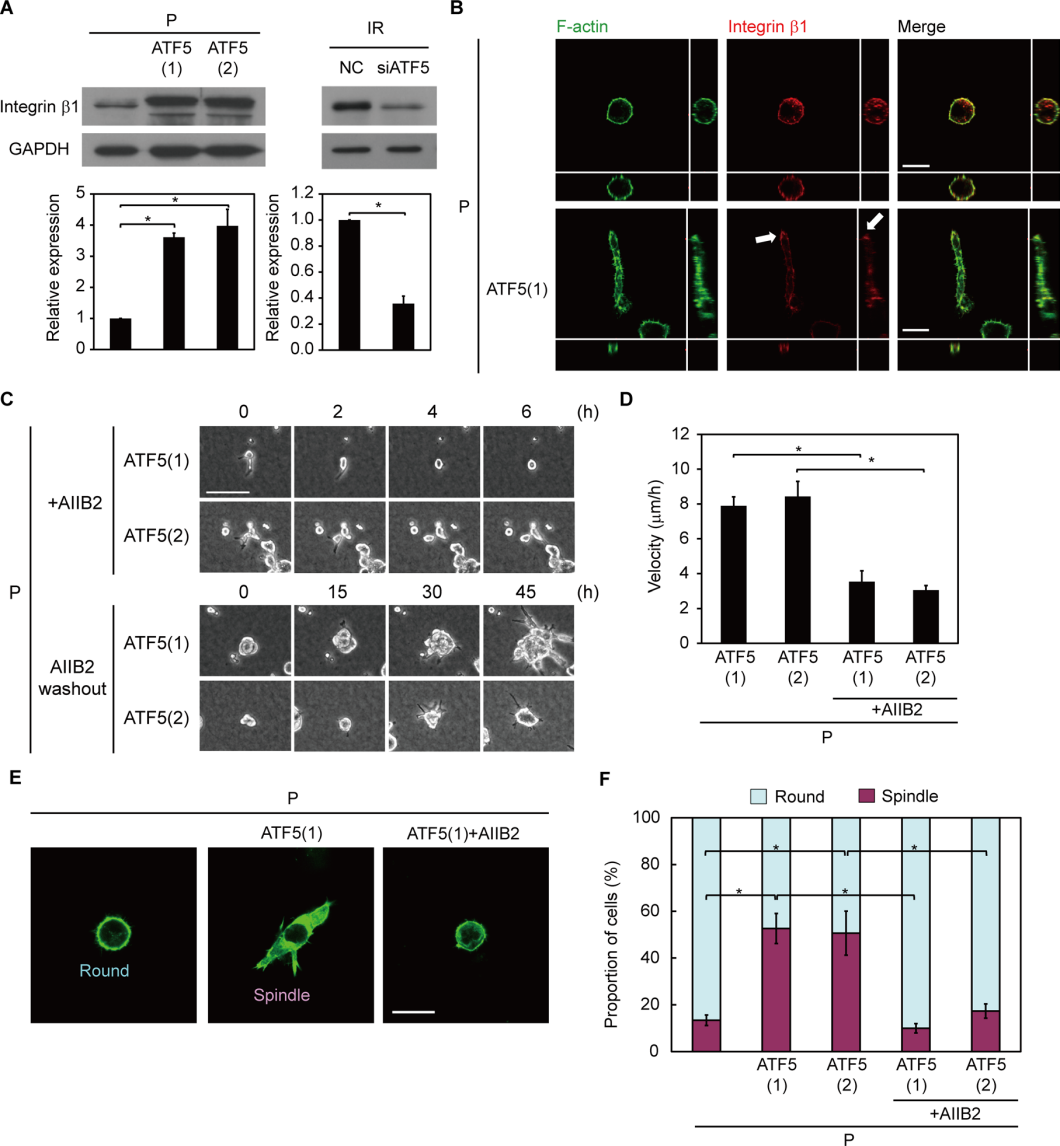
ATF5 induces integrin β1 expression, promoting invasiveness **(A)** Western blot of integrin β1 and GAPDH. The graph indicates the relative expression of integrin β1. P: subclonal A549 cells. P-ATF5(1), (2): subclonal P cells overexpressing ATF5. IR: P cells that survived irradiation. NC: negative control cells. siATF5: cells transfected with siRNA targeting ATF5. **(B)** Fluorescence images of F-actin and integrin β1. Cross-sectional views are shown. The arrows show the accumulation of integrin β1 at the tip of a protrusion. Bar = 20 μm. **(C)** Time-lapse phase-contrast images. The numbers indicate the time after the addition of AIIB2 or washout of AIIB2. +AIIB2: cells with AIIB2. AIIB2 washout: cells after washout of AIIB2. Bar = 100 μm. **(D)** Velocity of cell migration in C. Error bars = s.e.m. from 14 (P-ATF5(1) and (2)), 12 (P-ATF5(1)+AIIB2), or 15 (P-ATF5(2)+AIIB2) cells in 3 independent experiments. **(E)** Fluorescence images of F-actin. Round and spindle morphologies are displayed. Bar = 30 μm. **(F)** Proportion of cells in E. *P < 0.05. Error bars = s.e.m. from 3 independent experiments except D. The cells were cultured in collagen gel-overlay conditions.

We previously showed that invasiveness in IR cells is dependent on the activity of integrin β1 [6], an adhesion protein that connects the cell surface to extracellular matrices such as collagen [25]. Therefore, we examined whether ATF5 regulates invasiveness via integrin β1. The protein expression of integrin β1 was greater in P-ATF5(1) and (2) cells than in P and P-CAAX cells in collagen gel-overlay conditions (Figure 4A and Supplementary Figure S1J). In contrast, transient overexpression of ATF5 did not enhance integrin β1 expression (Supplementary Figure S8A). Furthermore, mRNA expression of integrin β1 was lower in P-ATF5(1) and (2) cells than in P cells (Supplementary Figure S8B). These results suggest that ATF5 does not enhance the transcription of integrin β1 mRNA and promotes protein expression of integrin β1 by its long-lasting, stable expression. In P and P-CAAX cells, integrin β1 was uniformly localized at the cell periphery, whereas integrin β1 accumulated at the tips of protrusions in P-ATF5(1) cells (Figure 4B and Supplementary Figure S1K). In addition, ATF5 downregulation in IR cells repressed integrin β1 expression in collagen gel-overlay conditions (Figure 4A). Next, we inhibited integrin β1 activity in cancer cells using the inhibitory antibody AIIB2 [26] and examined invasiveness. AIIB2 treatment repressed invasiveness in P-ATF5(1) and (2) cells, and washout after AIIB2 treatment restored invasiveness (Figure 4C, 4D and Videos S2, S3). We also quantified cells according to their morphology in collagen gel-overlay conditions. A large number of P-ATF5(1) and (2) cells exhibited spindle morphology, indicative of high invasiveness (Figure 4E, 4F). In contrast, P cells, P-CAAX cells, AIIB2-treated P-ATF5(1) cells, and AIIB2-treated P-ATF5(2) cells mainly exhibited round morphology, indicative of low invasiveness (Figure 4E, 4F and Supplementary Figure S1L, S1M). Collectively, these results indicate that ATF5 enhances invasiveness via integrin β1.

### ATF5 represses MRLC diphosphorylation

**Figure 5:**
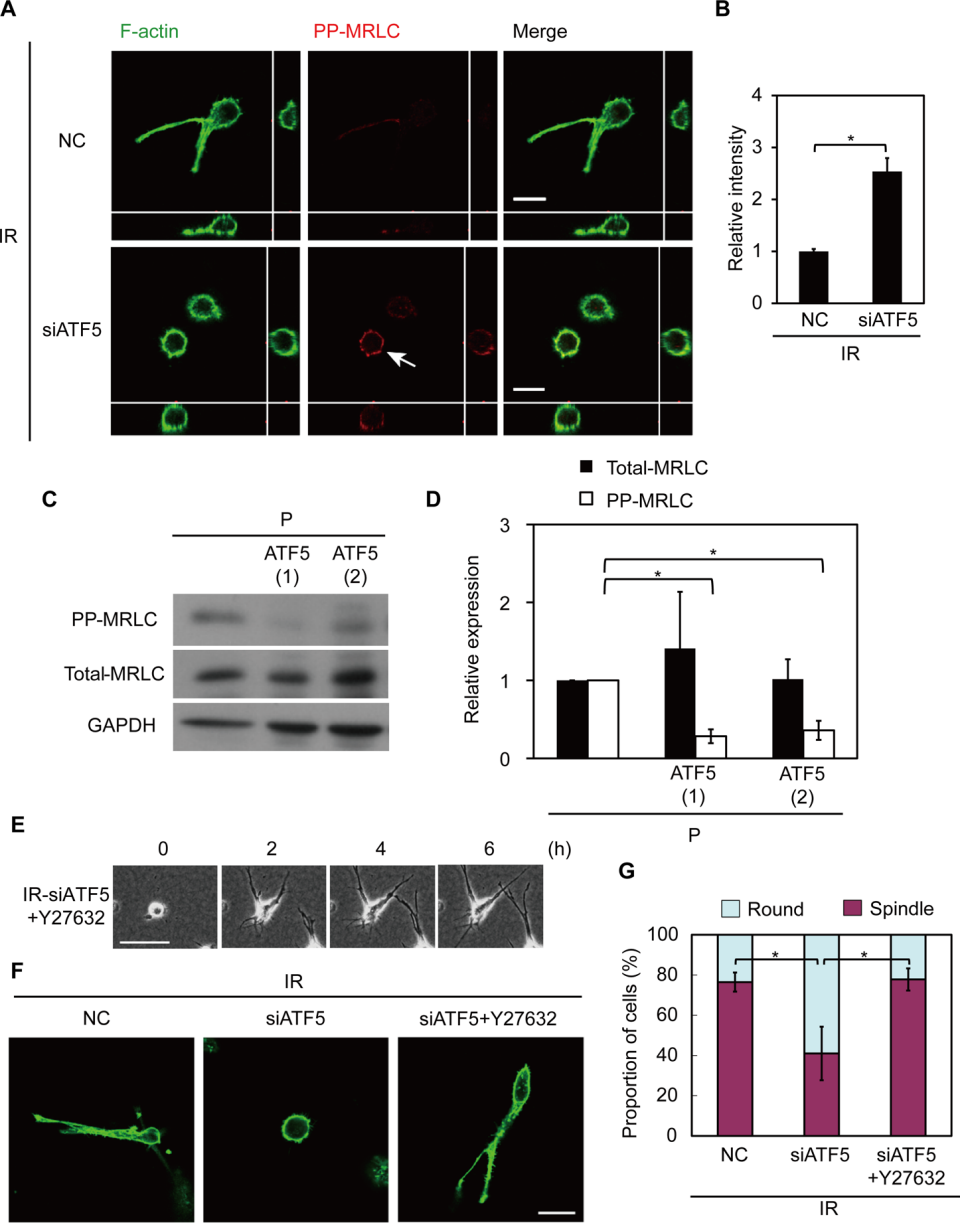
ATF5 perturbs the diphosphorylation of myosin regulatory light chain (MRLC), impairing invasiveness **(A)** Fluorescence images of F-actin and diphosphorylated MRLC (PP-MRLC). Cross-sectional views are shown. Arrow shows the accumulation of PP-MRLC.IR: subclonal A549 cells (P) that survived irradiation. NC: negative control cells. siATF5: cells transfected with siRNA targeting ATF5. Bar = 20 μm. **(B)** Relative intensity of PP-MRLC in A. Error bars = s.e.m. from 40 cells in 3 independent experiments. **(C)** Western blot of PP-MRLC, total-MRLC, and GAPDH in cells on a dish. P-ATF5(1), (2): subclonal P cells overexpressing ATF5. **(D)** Relative expression of proteins in C. Error bars = s.e.m. from 3 independent experiments. **(E)** Time-lapse phase-contrast images. The numbers indicate the time after the addition of Y27632. +Y27632: cells treated with Y27632. Bar = 100 μm. **(F)** Fluorescence images of F-actin. Bar = 30 μm. **(G)** Proportion of cells in F categorized as round or spindle shape. Error bars = s.e.m. from 4 (IR-NC and siATF5) or 3 (IR-siATF5+Y27632) independent experiments. *P < 0.05. The cells were cultured in collagen gel-overlay conditions except C.

We previously reported that the invasiveness of IR cells is dependent on MRLC dephosphorylation and that diphosphorylated MRLC (PP-MRLC) suppresses invasiveness in IR cells [7]. Therefore, we investigated the role of ATF5 in the diphosphorylation of MRLC. PP-MRLC was expressed at higher levels in IR cells in which ATF5 was downregulated than in control IR cells (Figure 5A, 5B). Total myosin regulatory light chain (total-MRLC) expression was not significantly affected by ATF5 downregulation (Supplementary Figure S9A, S9B). Furthermore, P-ATF5(1) and (2) cells expressed lower level of PP-MRLC than did P cells; total-MLRC expression was not significantly different (Figure 5C, 5D). P-CAAX cells had similar levels of PP-MRLC and total-MRLC expression as P cells (Supplementary Figure S1N). These results indicate that ATF5 suppresses the diphosphorylation of MRLC. We then treated ATF5-suppressed IR cells with Y27632 reagent, which inhibits MRLC diphosphorylation [27, 28] (Supplementary Figure S10A, S10B) and investigated the role of MRLC diphosphorylation in invasiveness. Most ATF5-suppressed IR cells exhibited low invasiveness and round morphology, but Y27632 treatment induced an invasive phenotype in these cells (Figure 5E–5G and Video S5). Thus, ATF5 induces invasiveness via the dephosphorylation of MRLC in IR cells.

We examined whether integrin β1 and MRLC regulate ATF5 and other proteins. Y27632 treatment did not affect ATF5 and integrin β1 expression in P cells (Supplementary Fig. S10A, B). In addition, AIIB2 treatment in IR cells did not affect ATF5 expression and MRLC diphosphorylation (Supplementary Figure S10C, S10D). These results suggest that integrin β1 and PP-MRLC do not regulate the expression of ATF5 or each other.

### ATF5 expression is correlated with poor prognosis

**Figure 6:**
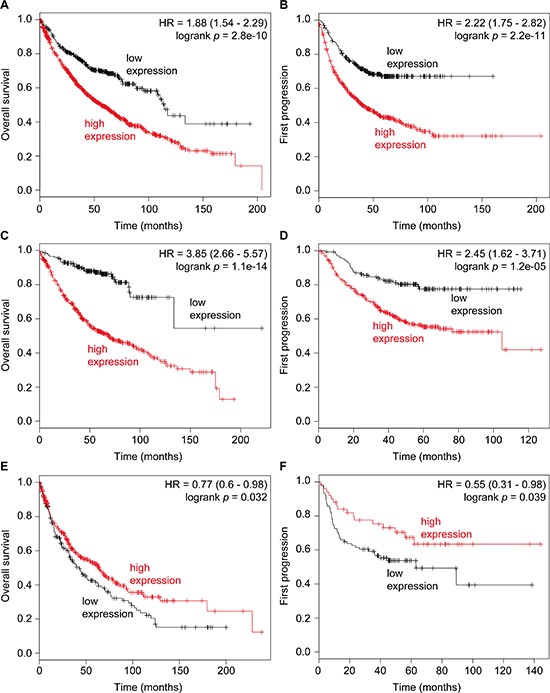
High expression of ATF5 is correlated with poor prognosis of lung cancer Kaplan–Meier survival curves showing the probability of overall survival **(A, C, E)** and first progression **(B, D, F)** in relation to the expression of ATF5 lung cancer patients. A, B: the data of overall non-small-cell lung cancer. C, D: the data of lung adenocarcinoma. E, F: the data of lung squamous cell carcinoma. n = 1432 (A), 787 (B), 719 (C), 461 (D), 525 (E), or 141 (F) patients.

We next investigated the relationship between the expression of ATF5 and the prognosis of lung cancer patients. Kaplan–Meier curves of overall survival and first progression in non-small-cell lung cancers were generated by *in silico* meta-analysis to reveal a positive correlation between ATF5 expression and poor prognosis in non-small-cell lung cancer and lung adenocarcinoma (Figure 6A, 6B, 6C, 6D). In contrast, ATF5 expression was negatively correlated with poor prognosis of lung squamous cell carcinoma (Figure 6E, 6F). These results suggest that high expression of ATF5 is a high risk factor for the progression of non-small-cell lung cancer including lung adenocarcinoma.

## DISCUSSION

**Figure 7:**
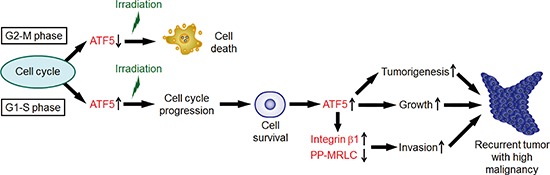
A model of tumor recurrence with high malignancy after irradiation In G1-S phase, cancer cells express ATF5, which enhances radioresistance by promoting cell cycle progression. On the other hand, in G2-M phase, cancer cells express low levels of ATF5 and display radiosensitivity. Cells that survived after irradiation express high levels of ATF5. ATF5 promotes tumorigenesis and cell growth. ATF5 also induces invasiveness via integrin β1 expression and inhibition of myosin regulatory light chain diphosphorylation (PP-MRLC).

In this study, we showed that ATF5 promotes radioresistance and malignancy in cancer cells (Figure 7). Furthermore, we showed that ATF5 expression was dependent on the cell cycle phase. In G2-M phases, cancer cells are radiosensitive because they express low levels of ATF5. In contrast, in G1-S phases, the cells are radioresistant because ATF5 expression induces cell cycle progression. In A549 cells, irradiation induces senescence and thereby leads to cell death [29]. In addition, cell cycle arrest induces senescence [30]. Therefore, ATF5 may enhance radioresistance by promoting cell cycle progression after irradiation and thereby preventing cell senescence. Furthermore, ATF5 perturbs the activity of p53 [16], which enhances senescence [30]. Thus, ATF5 may also perturb cell senescence by repressing p53 activity, resulting in enhanced radioresistance. We also showed that A549 cells that survive irradiation express high levels of ATF5, which enhances malignant phenotypes such as tumorigenesis, growth, and invasiveness in cancer cells. In addition, ATF5 promotes the expression of integrin β1 and represses MRLC diphosphorylation, thereby increasing invasiveness in cancer cells. A previous study showed that integrin β1 regulates cell-matrix adhesion [25]. Furthermore, diphosphorylated MRLC induces high cellular contractile force generated by actomyosin [31]. Thus, ATF5 might regulate invasiveness via cell-matrix adhesion regulated by integrin β1 and cellular contractile force modulated by MLRC phosphorylation. It has been unclear whether specific molecules regulate both radioresistance and malignant phenotypes, such as high proliferative ability and invasiveness, in cancer cells. In this study, we demonstrated that ATF5 enhances both radioresistance and malignancy in cancer cells after irradiation.

We showed that ATF5 perturbs MRLC phosphorylation in cancer cells. However, the detailed mechanisms of regulation in MRLC phosphorylation by ATF5 have not been identified. The possible mediators are Rho family proteins and specific kinases. RhoA, a Rho family protein, regulates MRLC phosphorylation by controlling Rho kinase (ROCK) activation [32, 28]. Furthermore, myosin light chain kinase (MLCK) phosphorylates MRLC by Ca^2+^ stimulation [33]. Thus, ATF5 might be regulating MRLC phosphorylation via RhoA-ROCK signaling and/or MLCK functions.

In this study, we showed that ATF5 enhances both radioresistance and malignant phenotypes in cancer cells. This double ability of ATF5 embodies the concept of “oncogenic resistance” [34, 35, 36]. According to this concept, some molecules not only enable cancer cells to resist some therapies, such as chemotherapy and radiotherapy, but also drive cancer progression. For example, BCL-ABL, Akt, and BCL-X_L_ enhance the resistance to paclitaxel, a commonly used chemotherapeutic reagent. These molecules also drive tumorigenesis [34]. We illustrated the concept in this study by showing that ATF5 induces both radioresistance and malignancy in cancer cells. We suggest that ATF5 functions as one of the key molecules in oncogenic resistance to radiotherapy.

We suggest that ATF5 is a possible therapeutic target for the treatment of malignant tumors in combination with radiotherapy. We demonstrated that ATF5 expression is correlated with poor prognosis of lung cancer. Furthermore, previous studies have indicated that repression of ATF5 induces cancer cell death and that ATF5 inhibition does not affect the viability of non-cancerous neural and breast cells [14, 15]. In addition, ATF5 induction in glioma cells enhances drug resistance [37]. These results support the idea that ATF5 is a possible therapeutic target for cancer treatment. From the results in this study, we suggest that ATF5 is a key regulator of cancer recurrence correlated with poor prognosis after radiotherapy. Therefore, inhibition of ATF5 may be an effective way to enhance radiosensitivity in cancer cells and prevent the recurrence and progression of cancer after radiotherapy.

## MATERIALS AND METHODS

### Cell culture and gene manipulation

The A549 human lung adenocarcinoma cell line was purchased from American Type Culture Collection (Manassas, VA). Subclonal A549 cells (P cells) and irradiation-tolerant P cells (IR cells) were established as previously reported [6]. P cells overexpressing the *ATF5* gene were obtained by transfecting cells with a pCMV6-XL5 vector carrying a human cDNA clone of ATF5 (OriGene, Rockville, MD) using Attractene Transfection Reagent (Qiagen, Hilden, Germany). P-ATF5(1) and (2) cells were established by subcloning P cells that overexpressed the *ATF5* gene. P cells were transfected with a phmAG1-H-Ras-CAAX vector [38] using Attractene Transfection Reagent and subcloned for the establishment of P-CAAX cells. For transient overexpression of the genes, we used Lipofectamine 2000 reagent (Invitrogen, Carlsbad, CA). The cells were cultured in Dulbecco's modified Eagle medium (DMEM; Sigma, St Louis, MO) containing 10% fetal bovine serum (Equitech-Bio Inc., Kerrville, TX or Biowest, Nuaillé, France) and 1% antibiotic/antimycotic solution (Sigma). The cells were incubated at 37°C in a humidified incubator with 5% CO_2_. siRNA and random RNA (for negative control) duplexes were synthesized using an *in vitro* transcription T7 kit (Takara, Otsu, Japan). The target sequences for the downregulation of specific genes were as follows: 5′-CAAAAATAAAACGAAACATTT-3′ for ATF5, 5′-CAAAGTTTGAAGAAATATACC-3′ for cyclin A2, and 5′-CAAGGAAAAGACATACTTAAG-3′ for cyclin E1 (sense sequence). The siRNA or random RNA duplexes were transfected into the cells using Lipofectamine RNAiMAX Reagent (Invitrogen). The cells transfected with siRNA or random RNA were used in experiments 3 days after transfection. A type I collagen gel (1.6 mg/mL, Cell matrix I-P; Nitta Gelatin, Osaka, Japan) was used for collagen gel-overlay culture and culture on collagen gels. The VenorGeM Mycoplasma Detection Kit (Minerva Biolabs, Berlin, Germany) was used to confirm the absence of mycoplasma contamination in all cells.

### Antibodies and reagents

Anti-ATF5 (1:7500–1:40,000; N-17, cat. #sc-46935; Santa Cruz Biotechnology, Inc., Santa Cruz, CA), anti-cyclin A2 (1:5000; cat. #4656; Cell Signaling Technology, Danvers, MA), anti-cyclin B1 (1:3000; cat. #4138; Cell Signaling Technology), anti-cyclin D1 (1:1000; cat. #2978; Cell Signaling Technology), anti-cyclin D3 (1:30,000; cat. #2936; Cell Signaling Technology), anti-cyclin E1 (1:60,000; cat. #4129; Cell Signaling Technology), anti-p-histone (1:2000; cat. #3377; Cell Signaling Technology), anti-integrin β1 (1:1000; cat. #610467; BD Bioscience, San Jose, CA), anti-GAPDH (1:10,000,000; cat. #1103016; Ambion, Foster City, CA), anti-diphosphorylated-myosin regulatory light chain (PP-MRLC; 1:100–1:300; cat. #3674; Cell Signaling Technology), anti-total-myosin regulatory light chain (total-MRLC; 1:100; cat. #3672; Cell Signaling Technology), HRP anti-goat IgG (1:20,000; cat. #305–035-003; Jackson ImmunoResearch Laboratories, Inc., West Grove, PA), HRP anti-rabbit IgG (1:10,000; cat. #7074; Cell Signaling Technology), and HRP anti-mouse IgG (1:10,000–1:200,000; cat. #70–6516; Bio-Rad, Hercules, CA) were used for western blotting. MFP488-phalloidin (1:500–1:1000; Mo Bi Tee, Göttingen, Germany) or Alexa Fluor 555-phalloidin (1:500; cat. #8953; Cell Signaling Technology) was used for F-actin staining. Anti-integrin β1 AIIB2 (350 ng/mL; Developmental Studies Hybridoma Bank at the University of Iowa, Iowa City, IA), anti-PP-MRLC (1:150), anti-total-MRLC (1:150), Alexa Fluor 594 anti-rat IgG (1:500; cat. #A-11007; Invitrogen), and Alexa Fluor 594 anti-rabbit IgG (1:500; cat. #A-11012; Invitrogen) were used for immunofluorescence staining. Propidium iodide (Sigma) was used for flow cytometry. Incubation with nocodazole (0.04 μg/mL; Sigma) for 12 h was used to synchronize cells at M phase. Incubation with mimosine (500 μM; Sigma) for 24 h was used to inhibit the G1/S cell cycle transition. Incubation with staurosporine (2 μM; Enzo Life Sciences, Plymouth Meeting, PA) for 4 h was used to induce apoptosis. Anti-integrin β1 AIIB2 (300 ng/mL) was used to inhibit integrin β1 activity. Y27632 (20 μM, Sigma) was used to inhibit MRLC diphosphorylation.

### Western blotting

To detect proteins from cells cultured on a dish, 0.5 × 10^5^ or 1.0 × 10^5^ cells were seeded on a culture dish (radius of 17.5 mm). One or two days after seeding, cell lysates were prepared using an improved method as previously reported [39]. To detect proteins from cells cultured in collagen gel-overlay conditions, 5 × 10^4^ cells were seeded on a culture dish (radius of 17.5 mm) filled with 500 μL of collagen gel. One day after seeding, 250 μL of collagen sol solution was poured on the cells and incubated for approximately 30 min at 37°C to induce gelation. The dish was then filled with culture medium and incubated at 37°C. After 1 day, cell lysates were prepared using an improved method as previously described [39]. Western blotting and quantification of protein expression were performed as previously reported [39, 40], but with blocking for 2 h to detect ATF5.

### Colony formation assay

Initially, 3.5 × 10^5^ cells (for non-treated P-3, P-ATF5(1), and P-ATF5 (2) cells), 1.3 × 10^5^ cells (for cells synchronized with nocodazole treatment), 2.6 × 10^5^ cells (for cells treated with mimosine and respective control cells), or 2.5 × 10^5^ cells (for cells transfected with siRNA or random RNA) were seeded in a 12.5-cm^2^ cell culture flask. Two days after seeding, cells were irradiated or non-irradiated at room temperature with a dose of 10 Gy. Cell cycles were synchronized with nocodazole treatment. Immediately after irradiation, the irradiated cells were dispersed with trypsin-EDTA. For mimosine-treated cells and the respective control cells, mimosine was added to the flask during the last minute of irradiation, and the cells were dispersed with trypsin-EDTA 24 h after irradiation. After dispersion, 1.0 × 10^3^ cells (for non-treated P-3, P-ATF5(1), and P-ATF5(2) cells), 5.0 × 10^3^ cells (for cells synchronized with nocodazole treatment), or 1.0 × 10^4^ cells (for cells treated with mimosine and the respective control cells) were reseeded on a 24-well plate. At the same time, non-irradiated cells were dispersed, and 100 cells were re-seeded on a culture dish (radius of 17.5 mm). The number of lump colonies of irradiated cells in a well and non-irradiated cells in a dish were counted 12 or 16 days after irradiation using a phase-contrast microscope (TE300; Nikon Instech Co., Tokyo, Japan; CKX41; Olympus, Tokyo, Japan) with a 4 × objective.

### Flow cytometry

Cells (2 × 10^5^) were seeded on a culture dish (radius of 17.5 mm). Two days after seeding, the cells were dispersed with trypsin-EDTA. The cells were washed with phosphate-buffered saline (PBS) and fixed with 70% iced ethanol with PBS for 30 min or more on ice. After fixation, the cells were incubated with 50 μg/mL propidium iodide and 50 μg/mL RNase A (Macherey-Nagel, Düren, Germany) in PBS for 30 min at 37°C. After incubation, the cell cycle phase was detected using a FACSCalibur cell analyzer (BD Biosciences).

### Cell growth assay

Cells (5 × 10^4^) were seeded on a culture dish (radius of 17.5 mm) filled with 500 μL of collagen gel. One day after seeding, 250 μL of collagen sol solution was poured on the cells and incubated for approximately 30 min at 37°C to induce gelation. The dish was then filled with culture medium and incubated at 37°C. After 1 day, cells were randomly imaged under a phase-contrast microscope (TE300; Nikon Instech Co.) with a 10× objective, and the colony size was determined by calculating the area of the colonies using ImageJ software (National Institutes of Health, Bethesda, MD). After 1, 2, or 3 day(s), the cells were treated with 0.1% collagenase-L (Nitta Gelatin) in PBS and incubated for 1 h at 37°C. After incubation, the cells were collected and suspended in 500 μL of trypsin-EDTA. The OD600 was calculated using an absorption spectrometer (SmartSpec Plus; Bio-Rad), and the cell number was determined according to the following formula: cell number = OD600 × 6.85 × 10^5^. Alternatively, cell number was directly counted by a counting chamber.

### Time-lapse imaging

A glass dish (radius of 12.5 mm) was filled with 500 μL of collagen gel, and 1.0 × 10^4^ cells were seeded on the gel. After 24 h, 250 μL of collagen sol solution was poured on the cell and incubated for approximately 30 min at 37°C to induce gelation. The dish was then filled with culture medium and incubated at 37°C. After 1 day, the dish was filled with medium and sealed with silicone grease to prevent changes in the pH of the medium. A phase-contrast microscope (TE300 or TS100; Nikon Instech Co.) with a 10× objective was used for time-lapse imaging. The dish with the cells was kept at 37°C in an acrylic resin box or on a thermo plate. Image-Pro software (Media Cybernetics Inc., Silver Spring, MD) or WraySpect software (Wraymer Inc., Osaka, Japan) was used to capture images every 5 min for time-lapse imaging. AIIB2 or Y27632 was added to the samples 24 h after the start of observation, and the observation was continued. After 24 h, the samples were washed with PBS, and the observation was continued. The velocity of cell migration was calculated by measuring the displacement of the cell center every 1 h using ImageJ software. The cells were selected randomly.

### Immunofluorescence staining

Cells (4 × 10^3^) were seeded on a glass dish (radius of 8.0 mm) filled with 100 μL of collagen gel. One day after seeding, 50 μL of collagen sol solution was poured on the cells and incubated for approximately 30 min at 37°C to induce gelation. The dish was then filled with culture medium and incubated at 37°C. After 1 day, cells were fixed with 4% formaldehyde in PBS and permeabilized with 0.5% Triton X-100 in PBS. Cells were blocked with 0.5% bovine serum albumin (for integrin β1 staining) or 0.5% skim milk (for PP-MRLC and total-MRLC staining) in PBS. Integrin β1, PP-MRLC, total-MRLC, and F-actin were stained with antibodies or a reagent, as described above. Fluorescence images were obtained using a confocal laser scanning microscope (C1 confocal imaging system; Nikon Instech Co.) with a 60× objective. The fluorescence intensities were analyzed using Image-Pro software (Media Cybernetics Inc.).

### qPCR

Cells (5 × 10^4^) were seeded on a culture dish (radius of 17.5 mm) filled with 500 μL of collagen gel. 1 day after seeding, 250 μL of collagen sol solution was poured on the cells and incubated for approximately 30 min at 37°C to induce gelation. The dish was then filled with culture medium and incubated at 37°C. After 1 day, cells were lysed with TriPure Isolation Reagent (Roche, Basel, Switzerland) for RNA extraction. Reverse transcription reaction was performed using ReverTra Ace qPCR RT Master Mix (TOYOBO, Osaka, Japan). qPCR was performed with DyNAmo ColorFlash SYBR Green qPCR Kit (Thermo Fisher Scientific Inc., Waltham, MA). Primers were as follows: β-actin (for internal control), 5′-GAGCCTCGCCTTTGCCGATCC-3′ (upper) and 5′-ACATGCCGGAGCCGTTGTCG-3′ (lower); and integrin β1, 5′-GACGCCGCGCGGAAAAGATG-3′ (upper) and 5′-GCACCACCCACAATTTGGCCC-3′ (lower).

### Roundness index analysis

Cells (1 × 10^4^) were seeded on a glass dish (radius of 12.5 mm) filled with 250 μL of collagen gel. One day after seeding, 125 μL of collagen sol solution was poured on the cells and incubated for approximately 30 min at 37°C to induce gelation. The dish was then filled with culture medium and incubated at 37°C with or without reagents. After 1 day, cells were fixed with 4% formaldehyde in PBS and permeabilized with 0.5% Triton X-100 in PBS. F-actin was stained with MFP-488 phalloidin, as described above. Fluorescence images were obtained using a confocal laser scanning microscope (C1 confocal imaging system; Nikon Instech Co.) with a 60× objective. The proportion of cells with round or spindle morphology was determined using the roundness index analysis, as previously reported [6].

### Luciferase reporter assay

A 24-well plate was filled with 100 μL of collagen gel, and 4.0 × 10^4^ cells were seeded on the gel. After 2 days, luciferase plasmids (300 ng/mL pCRE-Luc, 30 ng/mL pRL-TK, 1200 ng/mL pcDNA3) were transfected using Lipofectamine 2000 (Invitrogen). The cells were cultured in medium without antibiotics supplemented with 10% fetal bovine serum. After 1 day, the luciferase activities were detected using the Dual Luciferase Reporter Assay System (Promega, Madison, WI).

### Apoptosis assay

Cells (8 × 10^2^) were seeded on a glass dish (radius of 2.5 mm) filled with 25 μL of collagen gel. One day after seeding, 15 μL of collagen sol solution was poured on the cells and incubated for approximately 30 min at 37°C to induce gelation. The dish was then filled with culture medium and incubated at 37°C. After 1 day, apoptotic cells were detected using the GFP-Certified Apoptosis/Necrosis Detection System (Enzo Life Sciences).

### Experiments in mice

PBS-suspended cells (2.5 × 10^6^) were injected subcutaneously into the dorsal side of 7-week-old male BALB/cSlc-nu/nu mice (Japan SLC, Inc., Hamamatsu, Japan; 10 animals each). Macroscopic observation and tumor volume measurements were performed twice a week. The tumor volume was determined according to the following formula: tumor volume (mm^3^) = length (mm) × (width (mm))^2^/2. All animals were sacrificed 47 days after injection, and the tumor masses were dissected. Tumor tissues were fixed with 3.7% formaldehyde and embedded in paraffin. For each sample, 4 μm-thick sections were stained with hematoxylin and eosin. Mitotic cells were counted in 9 or 10 random high-power fields, and the average number of mitotic cells was determined. The animal experiments were strictly compliant with the animal care guidelines of Hokkaido University.

### Data analysis of patients

Kaplan–Meier curves were obtained by using the Kaplan–Meier plotter [41] at http://kmplot.com/analysis/. This software contains the databases of Cancer Biomedical Informatics Grid, Gene Expression Omnibus, and The Cancer Genome Atlas [41]. We used the “Auto select best cutoff” function, which computes the best performing threshold to determine high or low expression of ATF5.

### Statistics

The statistical significance of the experimental data was analyzed as follows. First, we determined whether the data met the normal distribution using the Kolmogorov–Smirnov test (*P* > 0.05 indicated the data met the normal distribution). When we analyzed the data set with normal distributions, we determined whether the variance of two data sets was significantly different (*P* < 0.05 indicated the variance was significantly different) using the F-test. For data sets with significantly different variances, we used Welch's *t*-test to determine the significance (*P*-value). On the other hand, for data sets without statistically different variances, we used Student's *t*-test to determine the significance (*P*-value). When we analyzed data sets with non-normal distributions, we determined the significance (*P*-value) using the Mann–Whitney *U*-test. We used sufficient sample sizes to evaluate the statistical significance in each experiment (at least three samples each).

## SUPPLEMENTARY FIGURES


