# Supplementary material for: Activating transcription factor 5 enhances radioresistance and malignancy in cancer cells
Source: Oncotarget. 2015 Feb 17;6(7):4602–14. doi: 10.18632/oncotarget.2912 (PMC4467102; doi:10.18632/oncotarget.2912)
Supplement: Supplementary file 1 [file oncotarget-06-4602-s001.pdf]

SUPPLEMENTARY FIGURES

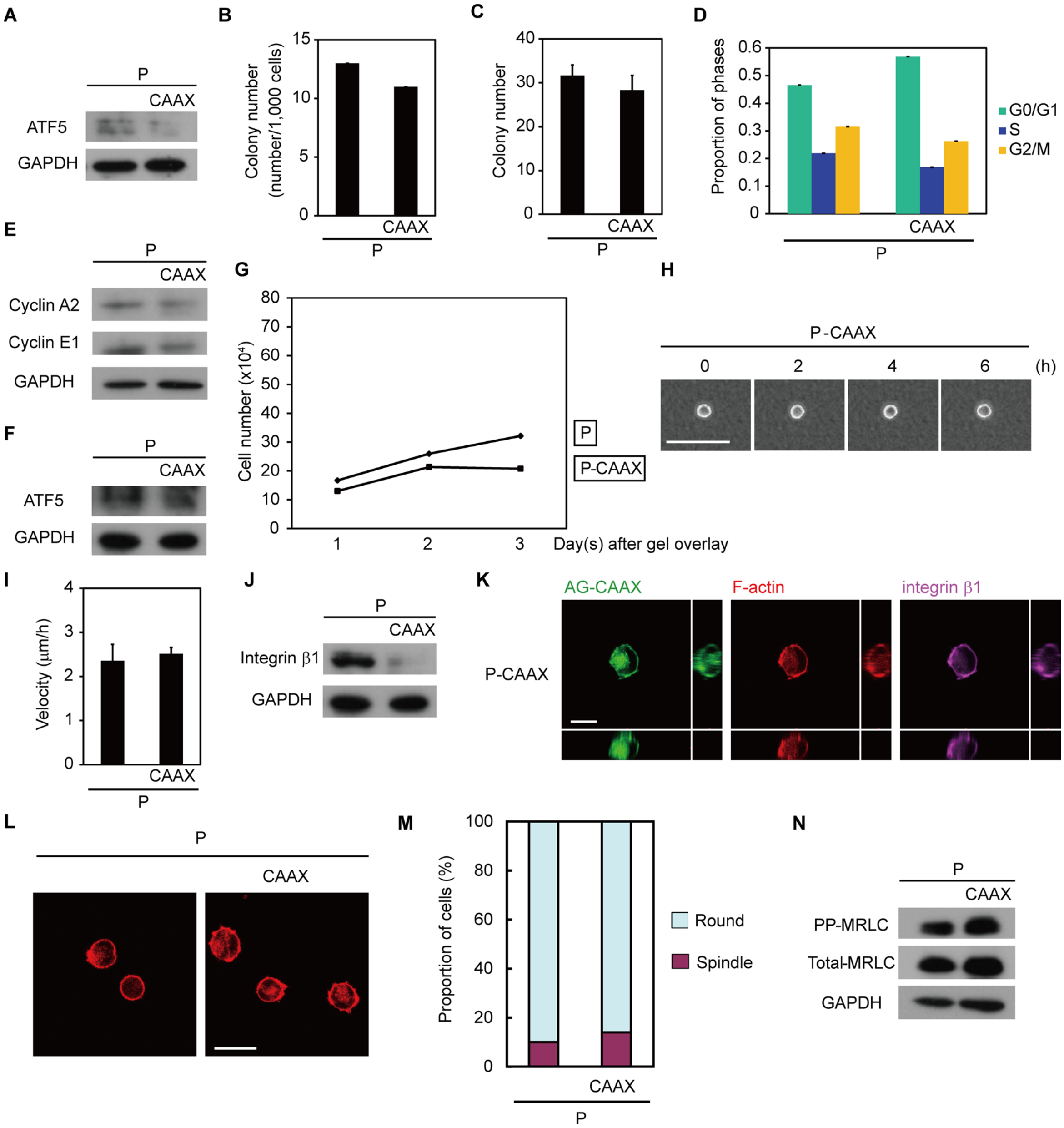

Supplementary Figure S1: Phenotypes of parental A549 cells (P) and subclonal P cells overexpressing AG-CAAX (P-CAAX) did not differ markedly. **(A)** Western blot of ATF5 and GAPDH. **(B)** Colony number 16 days after irradiation. **(C)** Colony number by non-irradiated cells on a culture dish 16 days after seeding. Error bars = s.e.m. from triplicate experiments. **(D)** Relative proportion of cells in the cell cycle phases determined by flow cytometry of cells stained with propidium iodide. **(E)** Western blot of cyclin A2, cyclin E1, and GAPDH. **(F)** Western blot of ATF5 and GAPDH. **(G)** Number of cells. The horizontal axis indicates the days after collagen gel-overlay. **(H)** Time-lapse phase-contrast images. The numbers indicate the time from the start of observation. Bar = 100 μm. **(I)** Velocity of migration in the cells. Error bars = s.e.m. from at least 4 cells. **(J)** Western blot of integrin β1 and GAPDH. **(K)** Fluorescence images of AG-CAAX, F-actin, and integrin β1. Cross-sectional views are shown. Bar = 20 μm. **(L)** Fluorescence images of F-actin stained by Alexa Fluor 555-phalloidin. Bar = 30 μm. **(M)** Proportion of cells in L categorized as round or spindle shape. **(N)** Western blot of PP-MRLC, total-MRLC, and GAPDH. F-M: The cells were cultured in collagen gel-overlay conditions.

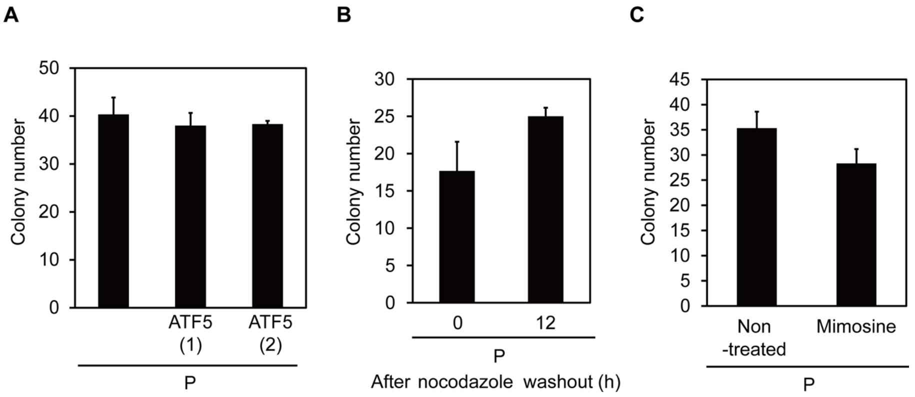

Supplementary Figure S2: Colony formation by non-irradiated cancer cells is not affected by ATF5 expression, synchronization, or mimosine treatment. **(A)** Colony number by non-irradiated cells on a culture dish 12 days after seeding. P: subclonal A549 cells. P-ATF5(1), (2): subclonal P cells overexpressing ATF5. Error bars = s.e.m. from triplicate experiments. **(B)** Colony number of non-irradiated P cells on a culture dish 12 days after seeding. The cells were seeded after cell cycle synchronization with nocodazole treatment and washout. The horizontal axis indicates the time after nocodazole washout. Error bars = s.e.m. from 3 independent experiments. **(C)** Colony number of non-irradiated P cells treated or not treated with mimosine 11 days after seeding on a culture dish. Error bars = s.e.m. from 3 independent experiments.

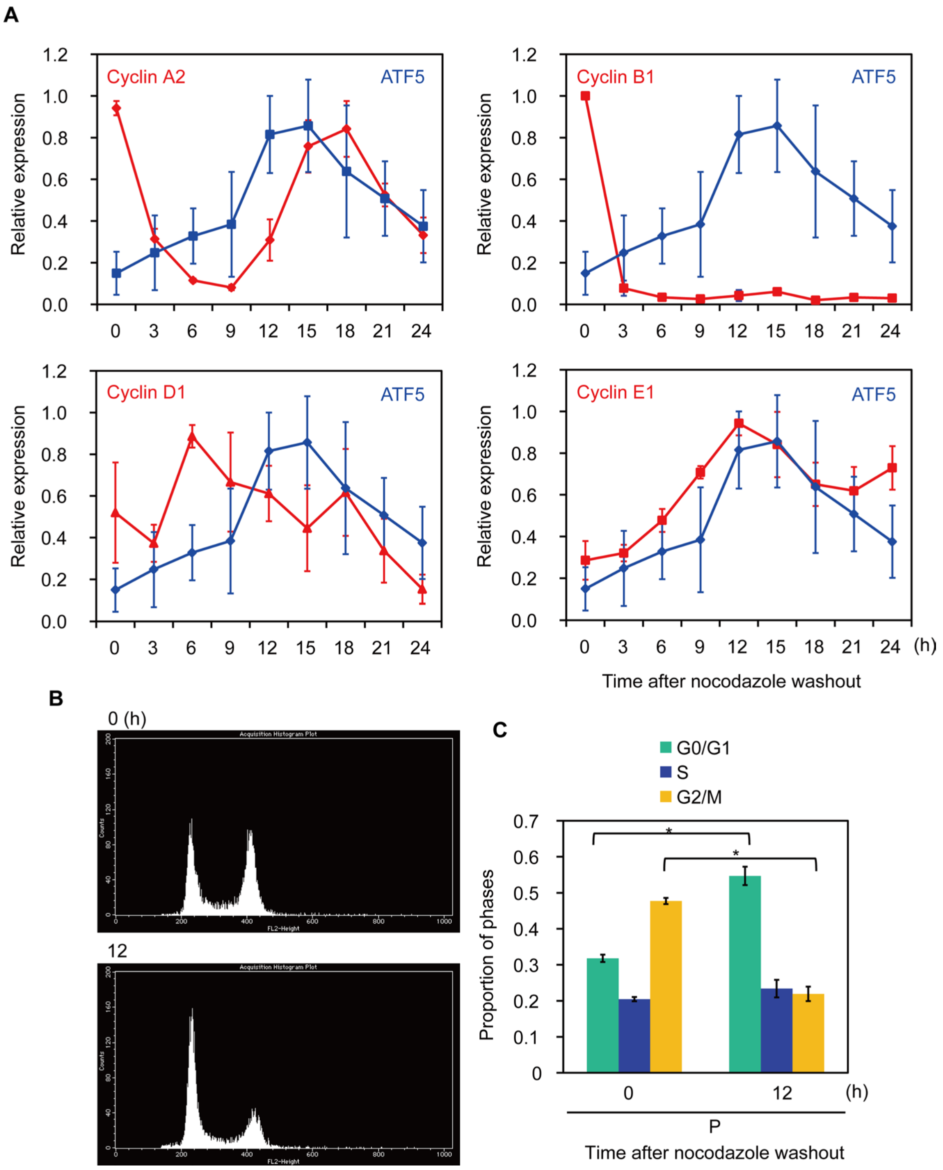

Supplementary Figure S3: ATF5 expression is dependent on the cell cycle phase in cancer cells. (A) Relative expression of ATF5, cyclin A2, cyclin B1, cyclin D1, and cyclin E1 in the subclonal A549 cells (P) shown in Figure 1c. The horizontal axis indicates the time after nocodazole washout. Error bars = s.e.m. from 3 independent experiments. **(B)** Flow cytometry of synchronized P cells stained with propidium iodide to detect the DNA content of cells on a culture dish. The numbers indicate the time after nocodazole washout. **(C)** Relative proportion of cells in the various cell cycle phases analyzed with flow cytometry in B. The numbers indicate the time after nocodazole washout. *$P < 0.05$. Error bars = s.e.m. from 3 independent experiments.

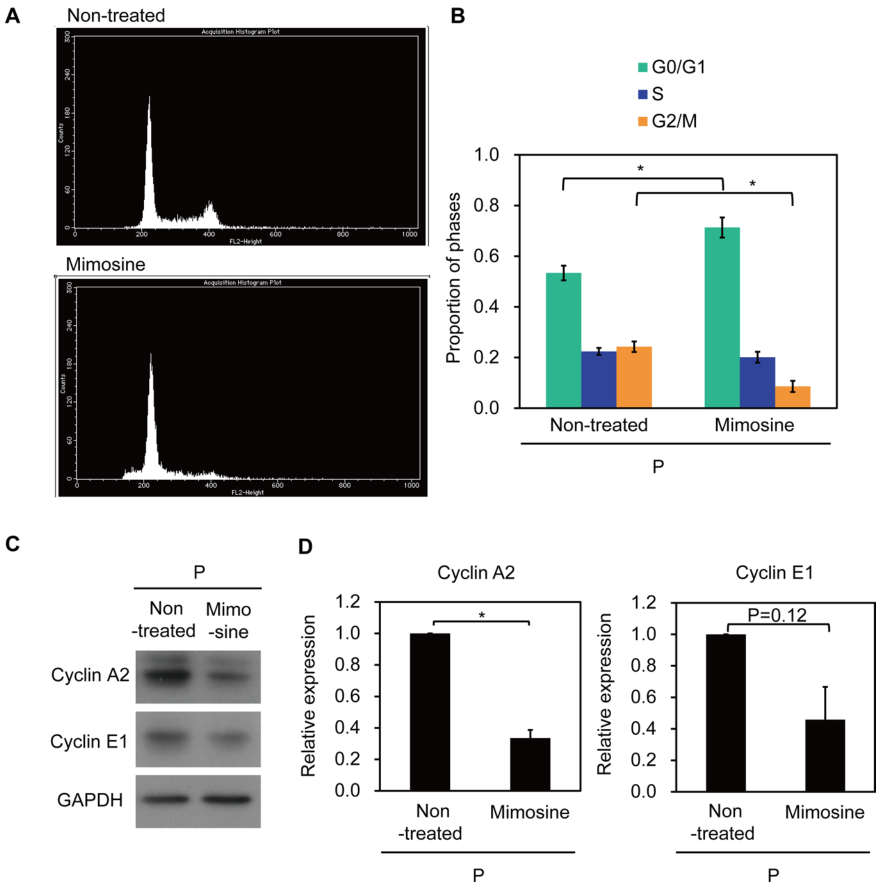

Supplementary Figure S4: Mimosine treatment blocks G1/S transition in cancer cells. (A) Flow cytometry of subclonal A549 cells (P) stained with propidium iodide to detect the DNA content of cells on a culture dish. P cells were treated or not treated with mimosine. **(B)** Relative proportion of cells in the various cell cycle phases determined with flow cytometry in A. $^*P < 0.05$. Error bars = s.e.m. from 3 independent experiments. **(C)** Western blot of cyclin A2, cyclin E1, and GAPDH in P cells treated or not treated with mimosine on a culture dish. **(D)** Relative expression of cyclin A2 and cyclin E1 analyzed as in C. $^*P < 0.05$. Error bars = s.e.m. from 3 independent experiments.

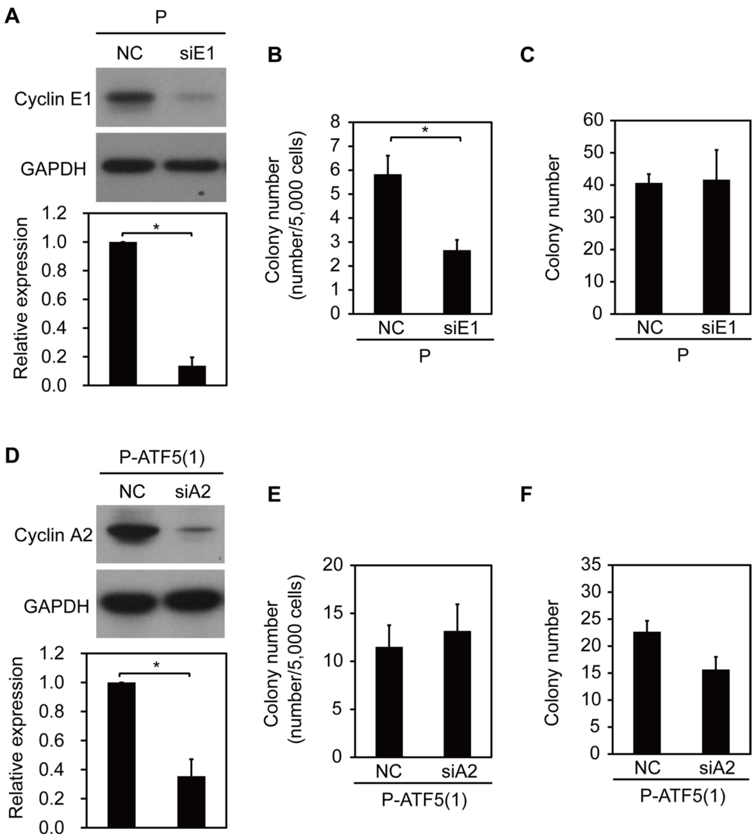

Supplementary Figure S5: Radioresistance in cancer cells is dependent on cyclin E1 and independent of cyclin A2. **(A)** Western blot of cyclin E1 and GAPDH in subclonal A549 cells (P) on a culture dish. The graph indicates the relative expression of cyclin E1. NC: negative control cells transfected with random RNA. siE1: cells transfected with siRNA targeting cyclin E1. *$P < 0.05$. Error bars = s.e.m. from 3 independent experiments. **(B)** Colony number of cells on a culture dish 12 days after 10-Gy irradiation. *$P < 0.05$. Error bars = s.e.m. from 3 independent experiments. **(C)** Colony number of non-irradiated cells on a culture dish 12 days after seeding. Error bars = s.e.m. from 3 independent experiments. **(D)** Western blot of cyclin A2 and GAPDH in subclonal P cell overexpressing ATF5 (P-ATF5(1)) on a culture dish. The graph indicates the relative expression of cyclin A2. siA2: cells transfected with siRNA targeting cyclin A2. *$P < 0.05$. Error bars = s.e.m. from 3 independent experiments. **(E)** Colony number of cells on a culture dish 12 days after 10-Gy irradiation. Error bars = s.e.m. from 3 independent experiments. **(F)** Colony number of non-irradiated cells on a culture dish 12 days after seeding. Error bars = s.e.m. from 3 independent experiments.

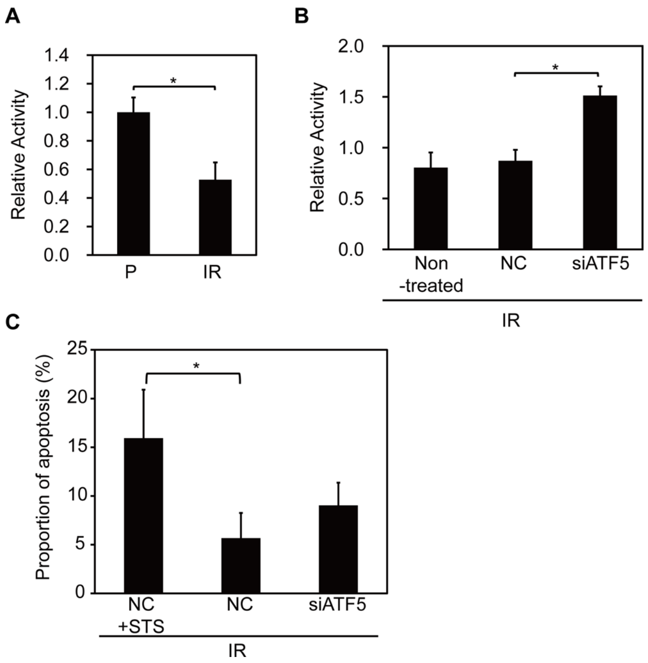

Supplementary Figure S6: ATF5 enhances pCRE activity but does not regulate apoptosis in cancer cells. (A) Luciferase reporter assay for the measurement of pCRE activity in A549 cells cultured on a collagen gel. P: subclonal A549 cells. IR: surviving P cells after 10-Gy irradiation. *$P < 0.05$. Error bars = s.e.m. from 3 independent experiments. **(B)** Luciferase reporter assay for the measurement of pCRE activity in IR cells cultured on a collagen gel. NC: negative control cells transfected with random RNA. siATF5: cells transfected with siRNA targeting ATF5. *$P < 0.05$. Error bars = s.e.m. from triplicate (non-treated and NC) or duplicate (siATF5) experiments. **(C)** Proportion of apoptotic IR cells cultured in collagen gel-overlay conditions. +STS: cells treated with staurosporine as an inducer of apoptosis. *$P < 0.05$. Error bars = S.D. from triplicate experiments.

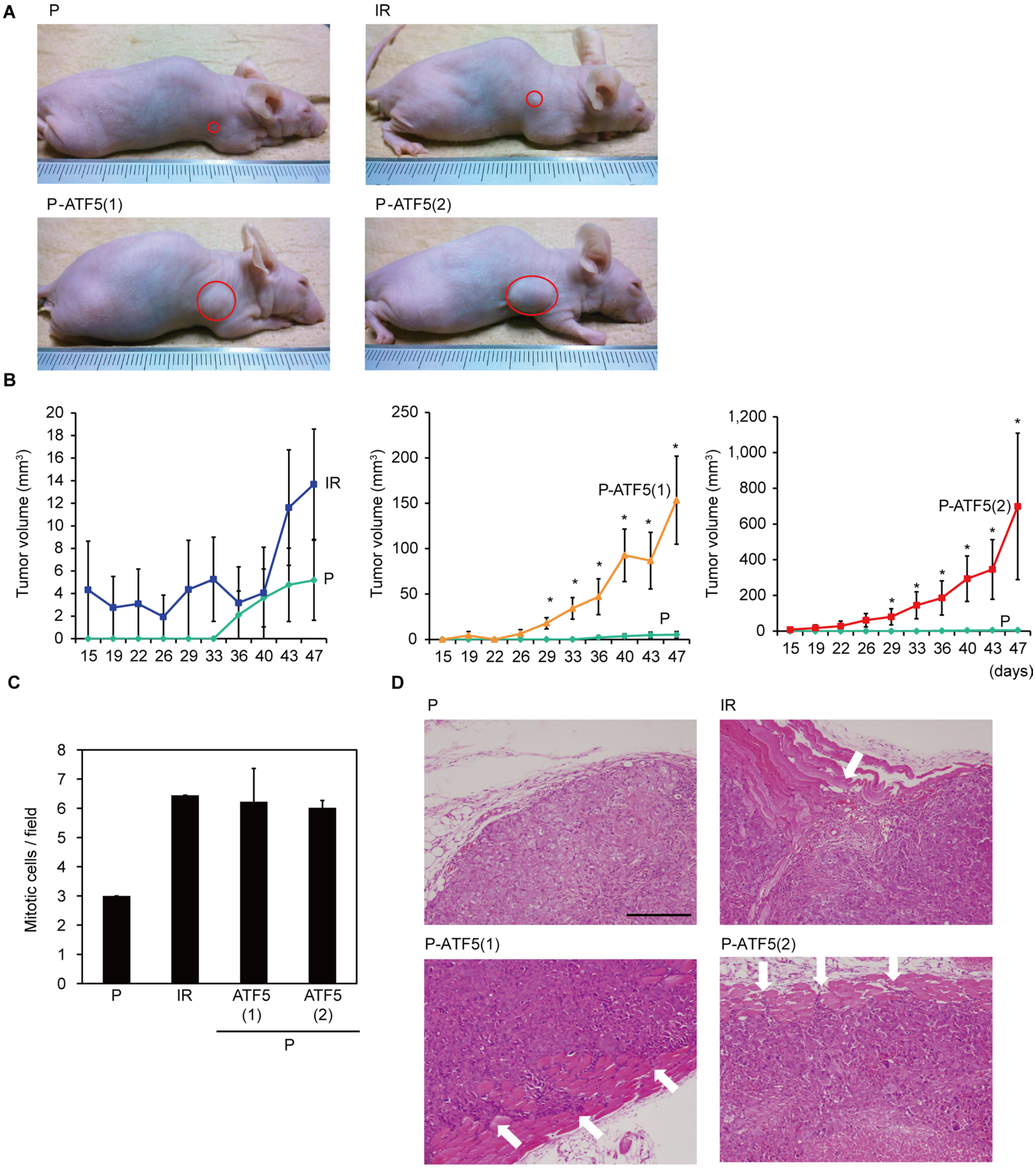

Supplementary Figure S7: Cancer cells that survive irradiation and cancer cells overexpressing ATF5 are highly malignant *in vivo*. **(A)** Nude mice with tumors generated by injected cancer cells. Red circles indicate the tumors. P: subclonal A549 cells. IR: surviving P cells after 10-Gy irradiation. P-ATF5(1), (2): subclonal P cells overexpressing ATF5. Each mark on the ruler indicates 1 mm. **(B)** Tumor volume determined from the experiment shown in A. The horizontal axis indicates the days after the injection of cancer cells. *$P < 0.05$. Error bars = s.e.m. from 10 mice in each condition. **(C)** Mitotic cells/field determined by histopathological observation of the tumors shown in A. Error bars = s.e.m. from 1 (P and IR) or 3 (P-ATF5(1) and (2)) tumors in each condition. **(D)** Histopathological images of tumors in the experiment shown in A. The arrows indicate the invasive front of the tumors. Bar = 200 μm.

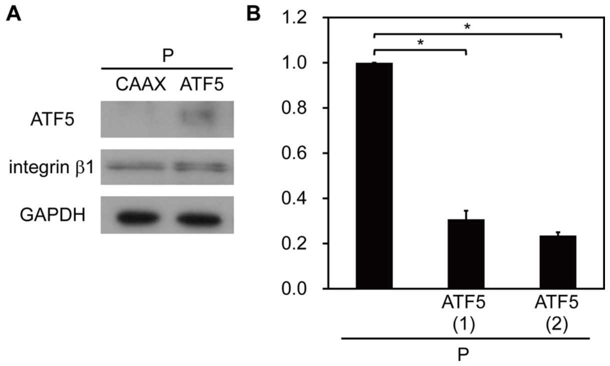

Supplementary Figure S8: ATF5 does not promote mRNA expression of integrin β1. **(A)** Western blot of ATF5, integrin β1, and GAPDH. P: subclonal A549 cells. P-CAAX: P cells transiently overexpressing AG-CAAX. P-ATF5: P cells transiently overexpressing ATF5. **(B)** qPCR analysis of integrin β1. P-ATF5(1), (2): subclonal P cells overexpressing ATF5. **P* < 0.05. Error bars = s.e.m. from 9 measurements in 3 independent experiments. The cells were cultured in collagen gel-overlay conditions.

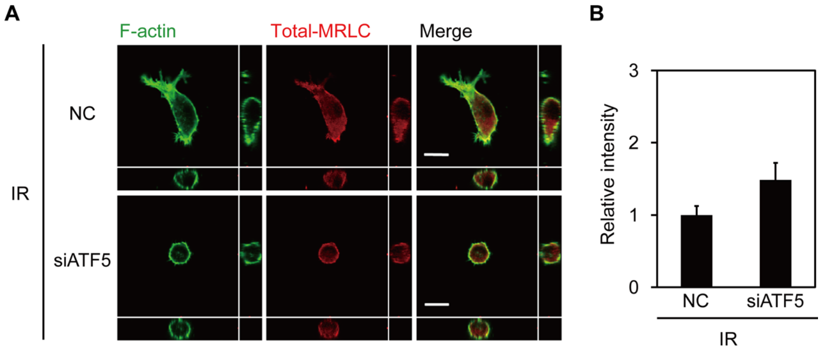

Supplementary Figure S9: ATF5 does not affect the expression of total myosin regulatory light chain in cancer cells. **(A)** Fluorescence images of F-actin and total myosin regulatory light chain (total-MRLC) in A549 cells cultured in collagen gel-overlay conditions. Cross-sectional views of the XZ and YZ directions are shown. IR: surviving subclonal A549 cells after 10-Gy irradiation. NC: negative control cells transfected with random RNA. siATF5: cells transfected with siRNA targeting ATF5. Bar = 20 µm. **(B)** Relative expression of total-MRLC in the cells shown in A. Error bars = s.e.m. from 10 cells.

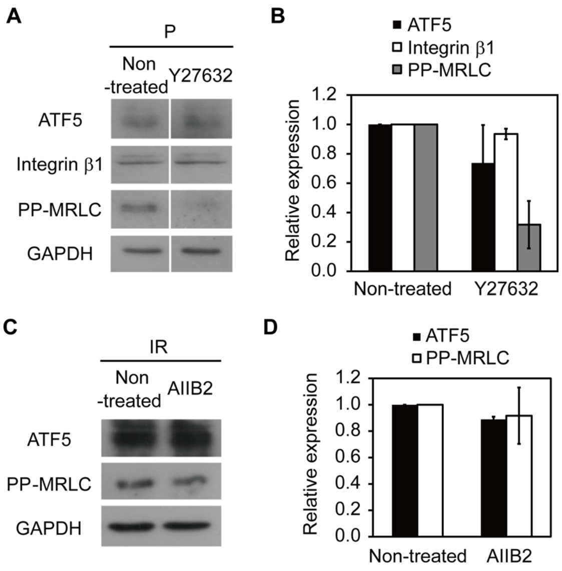

Supplementary Figure S10: Y27632 and AIIB2 treatment does not affect the expression of ATF5 and other proteins in cancer cells. **(A)** Western blot of ATF5, integrin β1, diphosphorylated myosin regulatory light chain (PP-MRLC), and GAPDH in cells cultured in collagen gel-overlay conditions. P: subclonal A549 cells. Y27632: cells treated with Y27632. **(B)** Relative expression of the proteins shown in A. Error bars = s.e.m. from 3 independent experiments. **(C)** Western blot of ATF5, PP-MRLC, and GAPDH in cells cultured in collagen gel-overlay conditions. IR: surviving P cells after 10-Gy irradiation. AIIB2: cells treated with AIIB2. **(D)** Relative expression of the proteins shown in C. Error bars = s.e.m. from 3 independent experiments.

SUPPLEMENTARY VIDEOS

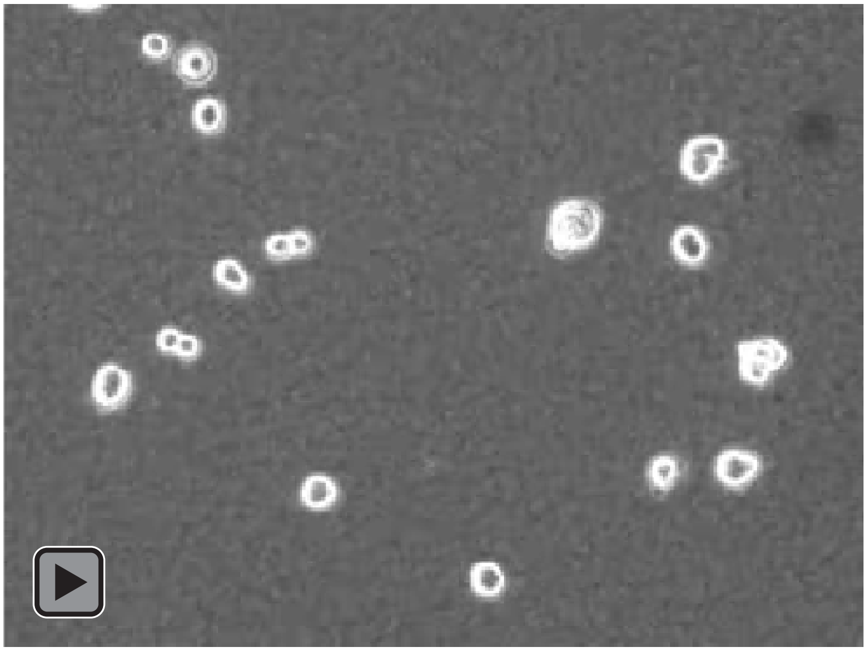

Supplementary Video S1: Time-lapse phase-contrast imaging of P cells cultured in collagen gel-overlay conditions. P cells were observed for 24 h. Video time, 1 s = real time, 150 min. Video width = 640 µm.

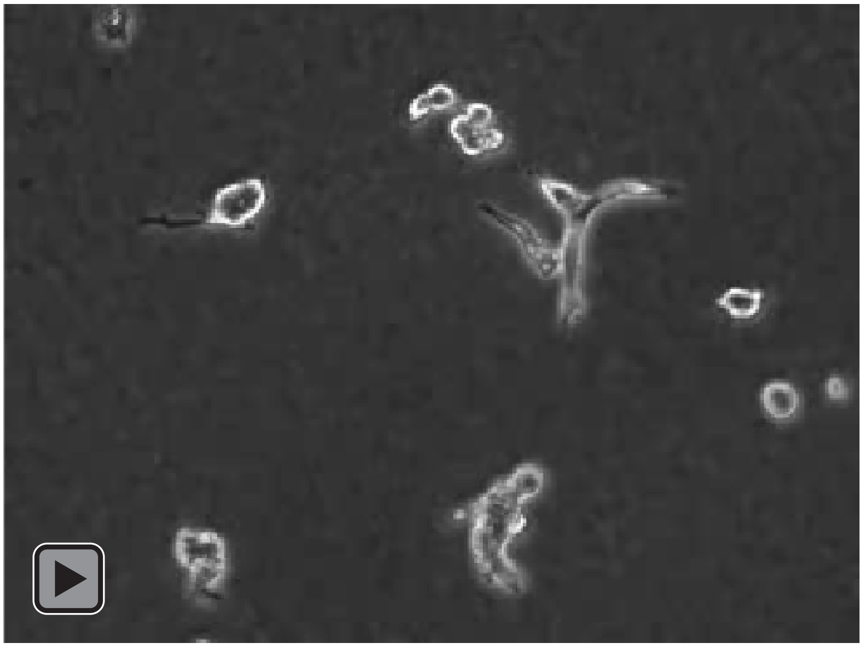

Supplementary Video S2: Time-lapse phase-contrast imaging of P-ATF5(1) cells cultured in collagen gel-overlay conditions. P-ATF5(1) cells were observed for 24 h (non-treated condition). The cells were then treated with AIIB2 and observed for another 24 h. After washout of AIIB2, the cells were observed for 48 h. Video time, 1 s = real time, 150 min. Video width = 640 µm.

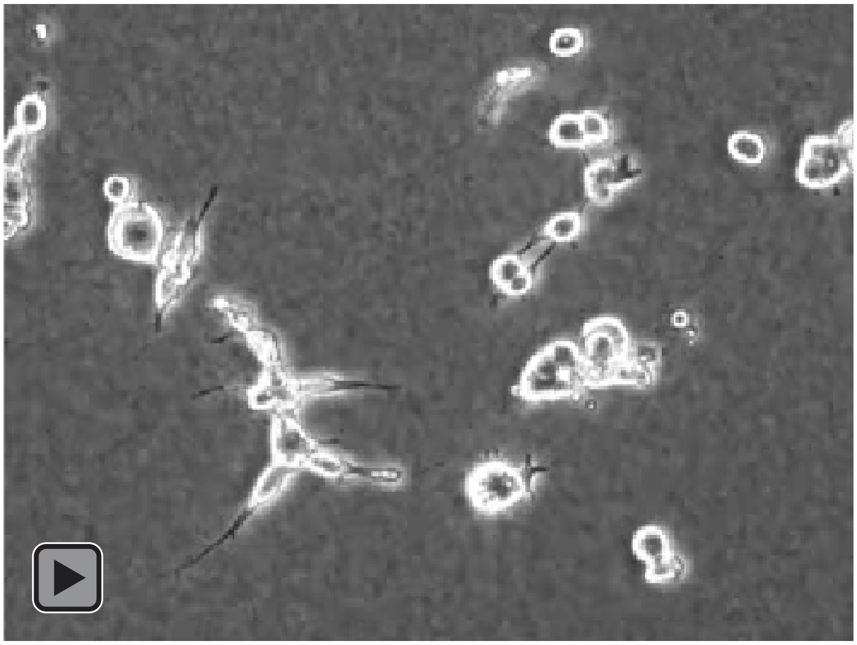

Supplementary Video S3: Time-lapse phase-contrast imaging of P-ATF5(2) cells cultured in collagen gel-overlay conditions. P-ATF5(2) cells were observed for 24 h (non-treated condition). The cells were then treated with AIIB2 and observed for another 24 h. After washout of AIIB2, the cells were observed for 48 h. Video time, 1 s = real time, 150 min. Video width = 640 µm.

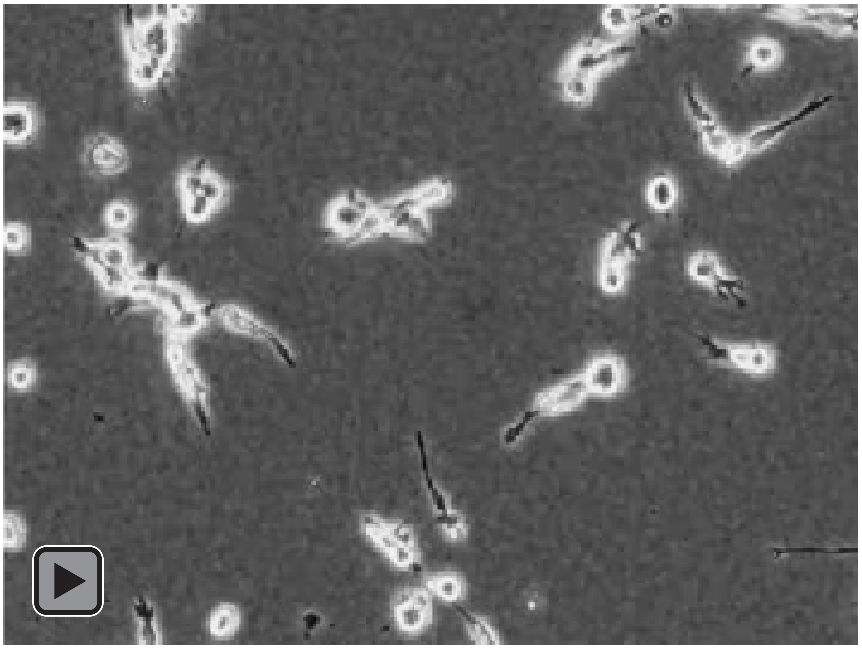

Supplementary Video S4: Time-lapse phase-contrast imaging of IR cells transfected with random RNA and cultured in collagen gel-overlay conditions. The cells were observed for 24 h. Video time, 1 s = real time, 150 min. Video width = 640 µm.

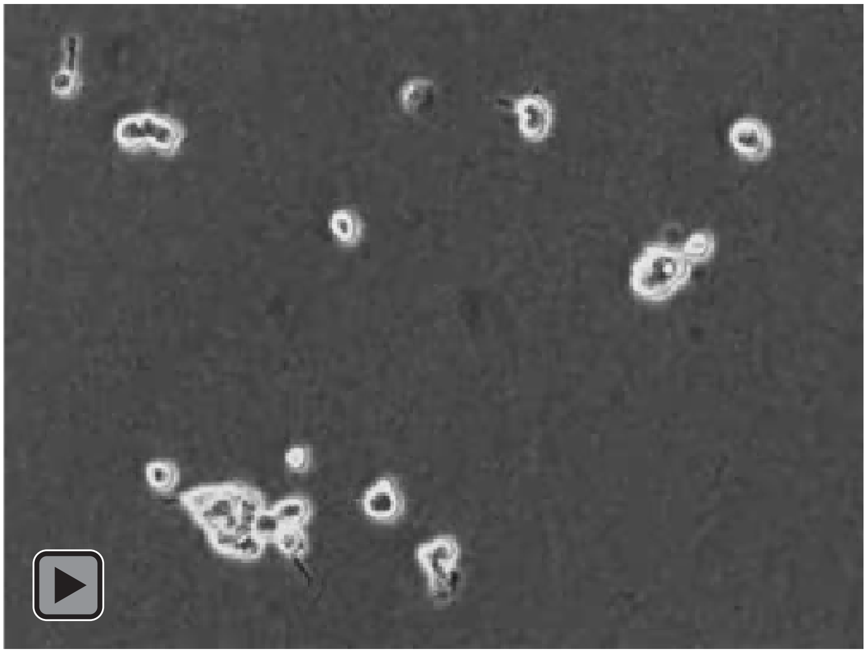

Supplementary Video S5: Time-lapse phase-contrast imaging of IR cells transfected with RNA targeting ATF5 and cultured in collagen gel-overlay conditions. The cells were observed for 24 h (non-treated condition). The cells were treated with Y27632 and observed for 24 h. Video time, 1 s = real time, 150 min. Video width = 640 µm.
